# IRE1-bZIP60 Pathway Is Required for *Nicotiana attenuata* Resistance to Fungal Pathogen *Alternaria alternata*

**DOI:** 10.3389/fpls.2019.00263

**Published:** 2019-03-19

**Authors:** Zhen Xu, Na Song, Lan Ma, Jinsong Wu

**Affiliations:** ^1^ Yunnan Key Laboratory for Wild Plant Resources, Kunming Institute of Botany, Chinese Academy of Sciences, Kunming, China; ^2^ University of Chinese Academy of Sciences, Beijing, China

**Keywords:** unfolded protein response, inositol-requiring enzyme 1 (IRE1), bZIP60, *Feruloyl-CoA 6ʹ-hydroxylase 1* (*F6ʹH1*), jasmonate (JA)

## Abstract

As an endoplasmic reticulum (ER) stress sensor, inositol-requiring enzyme 1 (IRE1) splices the bZIP60 mRNA, and produces an active bZIP60 transcription factor that regulates genes involved in the unfolded protein response (UPR) during ER stresses. This IRE1-bZIP60 pathway is conserved in plant species and recently implicated in plant-pathogen interaction. However, it is unclear whether this IRE1-bZIP60 pathway is involved in *Nicotiana attenuata* resistance to necrotic fungal pathogen, *Alternaria alternata*. In this study, transcriptional levels of chaperone protein genes, including *luminal binding protein* (*BiP*), *protein disulfide isomerase* (*PDI*), *calnexin 1-like* (*CNX 1-like*), and *calreticulin* (*CRT*), and genes involved in IRE1-bZIP60 pathway, were all significantly induced in *N. attenuata* leaves after *A. alternata* inoculation. Silencing *IRE1* or *bZIP60* led to *N. attenuata* plants more susceptible to *A. alternata*, which were associated with reduced gene expressions of *Feruloyl-CoA 6′-hydroxylase 1* (*F6′H1*), a gene encoding a key enzyme for phytoalexin scopoletin and scopolin biosynthesis. Further, electromobility shift assays (EMSA) indicated that bZIP60 protein of spliced form could directly bind to the promoter region of *F6′H1 in vitro*. JA signaling pathway is required for *N. attenuata* resistance to *A. alternata*. Interestingly, the fungus-elicited transcriptional levels of *BiP*, *PDI*, *CNX 1-like*, *CRT*, *IRE1*, and *bZIP60*(s) were all significantly decreased in JA-deficient or JA-insensitive plants. Meanwhile, those genes were significantly induced by methyl jasmonate (MeJA) when applied exogenously. However, the transcriptional levels of JA-regulated genes *allene oxide synthase* (*AOS*) and *lipoxygenease 3* (*LOX3*) were not affected in plants impaired with IRE1-bZIP60 pathway. Thus, it is concluded that IRE1-bZIP60 pathway is required for *N. attenuata* resistance to *A. alternata*, and JA signaling pathway plays an important role in the elicitation of chaperone protein genes and IRE1-bZIP60 pathway.

## Introduction

The endoplasmic reticulum (ER) is a major protein-folding organelle in cells (Ellgaard and Helenius, 2003; Trombetta and Parodi, 2003). During stresses, the protein-folding process is disturbed, thus a considerable amount of misfolded proteins are accumulated in the ER, leading to a situation known as ER stress (Gething and Sambrook, 1992; Vitale and Boston, 2008). The signaling network called unfolded protein response (UPR) plays an essential role in rescuing misfolded proteins to maintaining ER homeostasis, by enhancement of protein-folding and degradation of misfolded proteins, or by delaying new proteins synthesis. If UPR fails to mitigate ER stress, it will lead to programmed cell death and autophagy (Liu et al., 2012; Ruberti et al., 2015).

To facilitate folding and ensure quality of the produced protein, many factors including chaperones, folding factors, and enzymes, such as luminal binding protein (BiP), calnexin (CNX), calreticulin (CRT), and protein disulfide isomerase (PDI), are employed during ER stress (Siffroi-Fernandez et al., 2002; Freitas et al., 2016). The gene expressions of these factors, in many cases, also serve as molecular markers of the elicitation of UPR (Denecke et al., 1995; Klein et al., 2006).

There are two main branches of plant UPR to rehabilitate the ER capabilities, IRE1-bZIP60 and bZIP28-S1P/S2P pathways (Moreno and Orellana, 2011). IRE1-bZIP60 pathway is mediated by inositol-requiring enzyme 1 (IRE1) through a conserved mechanism. As a membrane kinase and ribonuclease, IRE1 serves as a stress sensor in eukaryotes by catalyzing the unconventional splicing of the mRNA of basic leucine zipper (bZIP) transcription factors when ER stress occurs. Plant bZIP60 has two stem loop structures, IRE1 recognizes these structures and cleaves two phosphodiester bonds, removes the coding region for a transmembrane domain (TMD), releasing the active form of bZIP60 (Deng et al., 2011). The transcription factor subsequently enters into the nucleus regulating the target genes for the restoration of ER proteostasis (Moreno and Orellana, 2011; Wan and Jiang, 2016). Another branch of plant UPR involves bZIP28. It is an ER membrane-associated transcription factor, migrates to Golgi during ER stress. bZIP28 is then cleaved off the transmembrane anchor by two Golgi-localized proteases S1P and S2P, and transported to the nucleus to regulate UPR-related genes (Wan and Jiang, 2016).

Heat, salt, drought, extreme osmotic and heavy metals stresses can activate the UPR in plant cell (Gao et al., 2008; Deng et al., 2011; Liu et al., 2011; Wang et al., 2011; Zhang et al., 2017; Li et al., 2018). There are also some reports linking pathogen attack and the activation of UPR (Tateda et al., 2008; Moreno et al., 2012; Ye et al., 2012; Sun et al., 2013). In *Arabidopsis*, *ire1a* mutant plants showed enhanced susceptibility to bacterial pathogen *Pseudomonas syringae* (Moreno et al., 2012). In addition, rice black-streaked dwarf virus P10 and potato virus X TGBp3 elicited the UPR responses in *N. benthamiana* (Sun et al., 2013; Ye et al., 2013). The IRE1/bZIP60 pathway suppressed systemic accumulation of potyviruses and potexviruses in *Arabidopsis* and *N. benthamiana* (Gaguancela et al., 2016). However, it is unclear whether UPR is involved in plant resistance to necrotrophic fungal pathogens.

The necrotrophic fungal pathogen *Alternata alternata* (tobacco pathotype) causes brown spot disease, which is one of the most common and destructive fungal diseases of *Nicotiana* species. In response to this fungal infection, phytoalexins scopoletin and scopolin and transcripts of their key enzyme gene *feruloyl-CoA 6′-hydroxylase 1* (*F6′H1*) are highly elicited in *N. attenuata* leaves around the attacked sites (Sun et al., 2014; Li and Wu, 2016). Phytoalexins are low-molecular mass antimicrobial secondary metabolites in response to pathogen infection, including camalexin, the major one in *Arabidopsis*, kauralexin, zealexin DIMBOA, and HDMBOA in maize, and scopoletin and capsidiol in *Nicotiana* species (Gnonlonfin et al., 2012). Scopoletin is a phenolic coumarin deriving from the phenylpropanoid pathway, playing an important role in *N. attenuata*-*A. alternata* interaction (Sun et al., 2014).

Jasmonate (JA) signaling pathway is usually associated with the defense against necrotrophic pathogens. Plants impaired with JA productions or JA perceptions are highly susceptible to *A. alternata* in *N. attenuata* (Sun et al., 2014). Importantly, phytoalexin scopoletin and scopolin biosyntheses are completely dependent on JA signaling, as their production and *F6*′*H1* transcripts are abolished in JA-deficient (irAOC) and JA-insensitive (irCOI1) plants (Sun et al., 2014, 2017). It is currently unknown whether IRE1-bZIP60 pathway is involved in the regulation of scopoletin and scopolin by JA signaling pathway.

Here, we investigated whether UPR was activated in *N. attenuata* plants in response to *A. alternata* inoculation. The role of IRE1-bZIP60 pathway in plant resistance was tested in plants silenced with either *IRE1* or *bZIP60 via* virus-induced gene silencing (VIGS), and the relation among IRE1-bZIP60 pathway, *F6*′*H1*, and JA signaling pathway was also explored in this study.

## Materials and Methods

### Plant and Fungal Material

Seeds of the *N. attenuata* were used as the wild-type (WT) genotype in all experiments. Stably transformed lines of irAOC and irCOI1 were previously generated (Paschold et al., 2007; Kallenbach et al., 2012) and used as plants that were silenced in the expression of *AOC* (the gene encoding the key enzyme of JA biosynthesis, *allene oxide cyclase*) and *COI1* (the gene encoding the JA-Ile receptor, *COI1*). Seed germination and plant growth were conducted as described by Krugel et al. (2002). Sterilized *N. attenuata* seeds were treated with 1:50 (v/v) liquid smoke (House of herbs, Passaic, NY, USA) and 1 mM gibberellic acid (GA3, www.sigmaaldrich.com) to break dormancy, and were sown on agar with Gamborg B5. After 10 days, seedlings were planted into soil in Teku pots for another 10 days, seedlings of similar size were then transferred into 1-L pots in greenhouse with day/night temperatures of 25/19°C. Plants around 35-day-old before bolting were used for experiments.

*Alternaria alternata* were grown and used for inoculation as described (Sun et al., 2014). In brief, the source-sink transition leaves (0 leaves) were detached and inoculated with 4 PDA plugs (3 mm diameter) containing actively growing hyphae of *A. alternata*, which had been grown on PDA plates for 6–8 days, and then placed in a transparent 12 × 12 cm square Petri dish with 100% humidity at 25°C with a 16-h light/8-h dark cycle. Detached leaf without PDA plugs was served as mock control. Lesion diameters were measured after inoculation for 5–7 days.

### Generation of VIGS Plants

Specific cDNA fragments of the *bZIP60*, *IRE1a,* and *IRE1b* were amplified by primers (XZ97 and XZ98 for bZIP60, XZ159 and XZ160 for *IRE1a*, XZ163 and XZ164 for *IRE1b*; all primers used in this study are included in Supplementary Table 1) and cloned into pTV00 respectively (Ratcliff et al., 2001). *Agrobacterium tumefacien*s GV3101 carrying above constructs were combined with those cells carrying pBINTRA, and then were inoculated into 3-week-old *N. attenuata* leaves, generating *bZIP60*-, *IRE1a*-, and and *IRE1b*-silenced plants (VIGS bZIP60, VIGS IRE1a, and VIGS IRE1b plants). To monitor the progress of VIGS, *phytoene desaturase* (*PDS*) was silenced, which would lead to a visible bleaching of tissues 2–3 weeks after inoculation (Saedler and Baldwin, 2004; Wu et al., 2008). When the leaves of *PDS*-silenced plants began to bleach, the source-sink transition leaves of VIGS plants and empty vector-inoculated plants (EV plants) were selected for further experiments. Around 25 plants were inoculated for each construct, and all VIGS experiments were repeated another two times.

### Real-Time PCR Assay

Total RNA of five biological-replicate samples was extracted from a 1.5 × 1.5 cm^2^ area of leaf lamina with the inoculation site at the center. cDNA was synthesized from 500 ng of total RNA with reverse transcriptase (Thermo Scientific, http://www.thermoscientifcbio.com). Real-time PCR was performed as described (Sun et al., 2014) on a CFX Connect qPCR System (Bio-Rad) with iTaq Universal SYBR Green Supermix (Bio-Rad) and gene-specific primers according to the manufacturer’s instructions. The transcript abundance of *Actin II* was not altered in leaves either inoculated with *A. alternata* at 1 or 3 dpi or treated with MeJA and thus was used as an internal standard (Xu et al., 2018). All primers used in this study are included in Supplementary Table 1.

### Electromobility Shift Assays (EMSA)

The full-length coding sequence of bZIP60 (spliced form) was cloned in frame into the E*coR* I-X*ho* I sites of the pET28a (+), His- bZIP60(s) were expressed and purified with Ni-NTA agarose (QIAGEN). Biotin-labeled probes FBP1 (5′-aattttttttACGTaaatgattttcttttaccTGAAAAtttcccttga-3′), FBP2 (5′-taaggggcgTGAAAAgttcaaagt-3′), and FBP3 (5′-ttttgcactCAGTCAggctaaaatcg-3′) were designed from *F6′H1* promoter and synthesized from Sangon Biotech (Shanghai). The detection of the binding of the recombinant protein and the probes (300 ng of recombinant protein and 30 ng labeled probe) was carried out with a chemiluminescent EMSA kit (Beyotime Biotechnology) according to the protocol suggested by the manufacturer.

### Methyl Jasmonate (MeJA) Treatments

MeJA treatment is the most commonly used means of eliciting JA-regulated responses in plants; the chemical will be quickly de-methylated into jasmonic acid by plant MeJA-esterase when applied (Wu et al., 2008). A solution of 1 mM MeJA^1^ was prepared by distilled water, and sprayed directly on 0 leaves of 35-day-old rosette staged *N. attenuata* plants until run off with a fine mist according to Xu et al. (2018). The treated plants were immediately covered with a plastic bag, and harvested after 1, 3, 6, and 12 h. Distilled water was sprayed and used as a control treatment.

### Accession Numbers

Sequence data from this article can be found in the GeneBank data library under accession numbers: XM_019373376.1 (*luminal binding protein*); XM_019393813.1 (*protein disulfide isomerase*); XM_019379334.1 (C*alnexin 1-like*); XM_019387223.1 (*Calreticulin*); XM_019409728.1 (*IRE1a*); XM_019389310.1 (*IRE1b*) and XM_019396471.1 (*bZIP60*); AY133805.1 (AtbZIP60); NM_127306.4 (AtIRE1a); and NM_001203454.1 (AtIRE1b).

### Statistical Analyses

Statistical differences in gene expression between mock and fugus infection were analyzed with the Student’s t-test (*p* < 0.05). The lesion diameter was analyzed with Student’s t-test (*p* < 0.05). All analyses were performed using SPASS statistical software.

## Result

### Elicitation of Chaperone Protein Genes and Genes Involved in IRE1-bZIP60 Pathway in *N. attenuata* After *A. alternata* Inoculation

**Figure 1:**
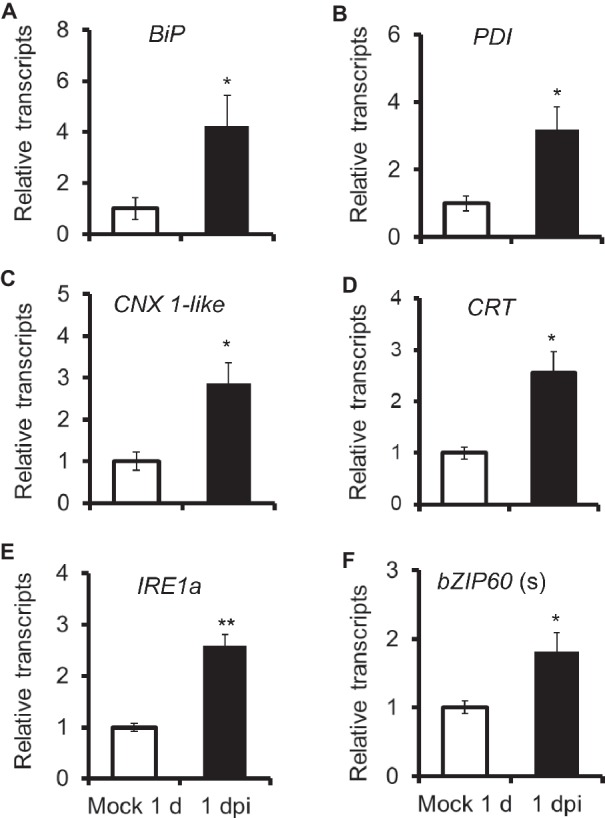
Elicitation of chaperone protein genes and genes involved in IRE-bZIP60 pathway after *A. alternata* inoculation. *BiP* **(A)**, *PDI* **(B)**, *CNX 1-like* **(C)**, *CRT* **(D)**, *IRE1a* **(E)**, and *bZIP60*(s) **(F)** transcripts were measured by real-time PCR in source-sink transition leaves treated with mock infection of with *A. alternata* at 1 day post inoculation (dpi). All transcriptional levels were normalized with a housekeeping gene *Actin II*. Values are means ±SE for five biological replicates. Asterisks indicate the level of significant difference between mock and infected leaves (Student’s *t*-test: *, *p* < 0.05; **, *p* < 0.01, *n* = 5). All those experiments were repeated three times.

To test whether unfolded protein response was involved in *N. attenuata* resistance to *A. alternata*, we detected gene expression of chaperone proteins, which were usually up-regulated to accelerate protein folding during ER stress. All four chaperone protein genes tested in this study, *BiP*, *PDI*, *CNX 1-like*, and *CRT*, were significantly induced after 1 day post inoculation (dpi) (Figure 1). This result indicated that ER stress occurred in *N. attenuata* plants after *A. alternata* infection and host plants tried to alle*via*te this stress by activation of UPR.

Next, we investigated transcriptional level of genes involved in IRE1-bZIP60 pathway. The *N. attenuata* genome comprises two *IRE1*s, *IRE1a* (XM_019409728.1), and *IRE1b* (XM_019389310.1), and one bZIP60 (XM_019396471.1). Both IRE1a and IRE1b shared 47% amino acid identity (Supplementary Data 1). Specific primers were designed to distinguish the *bZIP60* spliced form (*bZIP60* (s)) and unspliced form (*bZIP60* (u); Supplementary Data 3). Our results indicated that *IRE1a* and *bZIP60* (s) were both significantly elicited in *N. attenuata* plants at 1 dpi (Figure 1).

### IRE1-bZIP60 Pathway Is Required for *N. attenuata* Resistance to the Fungal Pathogen *A. alternata*

**Figure 2:**
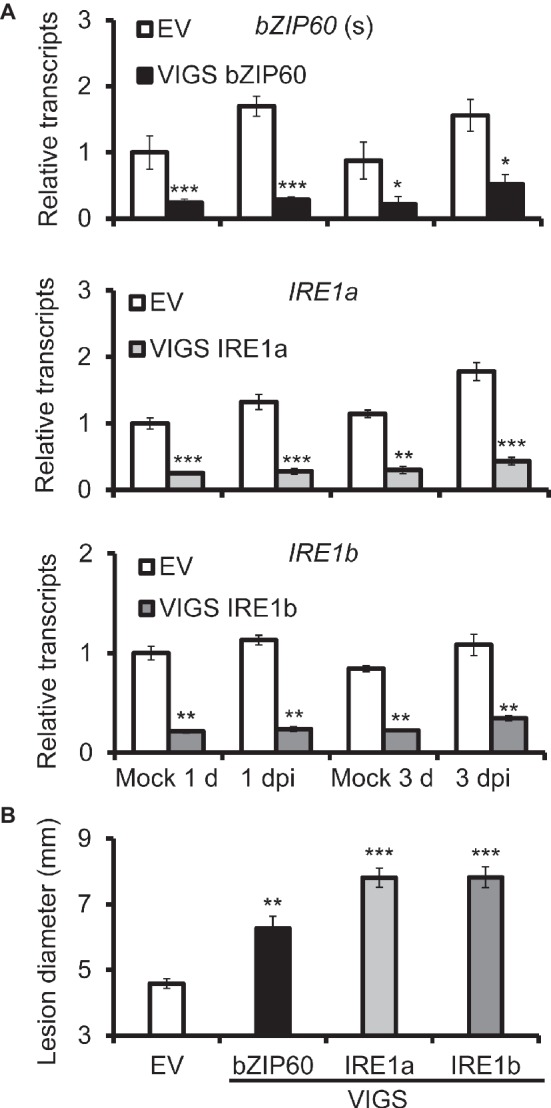
Silencing *bZIP60 and IRE1*s led to plants susceptible to *A. alternata*. **(A)** Mean (±SE) *bZIP60*(s), *IRE1a*, and *IRE1b* transcripts were measured by real-time PCR in six biological replicates of source-sink transition leaves of EV, VIGS bZIP60, VIGS IRE1a, and VIGS IRE1a plants at 1 and 3 dpi (*n* = 6). **(B)** Mean (±SE) diameter of necrotic lesions of 20 biological replicates of source-sink transition leaves of EV, VIGS bZIP60, VIGS IRE1a, and VIGS IRE1b plants at 6 dpi (*n* = 20). Asterisks indicate the level of significant differences between EV and VIGS plants with the same treatments (Student’s *t*-test: *, *p* < 0.05; **, *p* < 0.01; ***, *p* < 0.005). All those experiments were repeated four times.

To study the role of IRE1-bZIP60 pathway in *N. attenuata* resistance to *A. alternata*, *IRE1a*, *IRE1b*, and *bZIP60* were silenced *via* virus-induced gene silencing (VIGS) individually. The transcriptional levels of *IRE1a* were reduced by more than 80% in plants transformed with *IRE1a*-silenced construct (VIGS IRE1a) when compared with plants transformed with empty vector (EV) at 1 dpi, and decreased by 66% at 3 dpi, suggesting that *IRE1a* was successfully silenced in VIGS IRE1a plants (Figure 2A). Similarly, VIGS IRE1b plants and VIGS bZIP60 plants were also successfully generated (Figure 2A). Notably, significantly bigger lesions were observed in source-sink transition leaves of plants silenced with *IRE1a*, *IRE1b*, or *bZIP60* at 6 dpi when compared with EV plants (Figure 2B, Supplementary Data 4). These results indicate that plants impaired with IRE1-bZIP60 pathway are more susceptible to *A. alternata* than EV plants.

**Figure 3:**
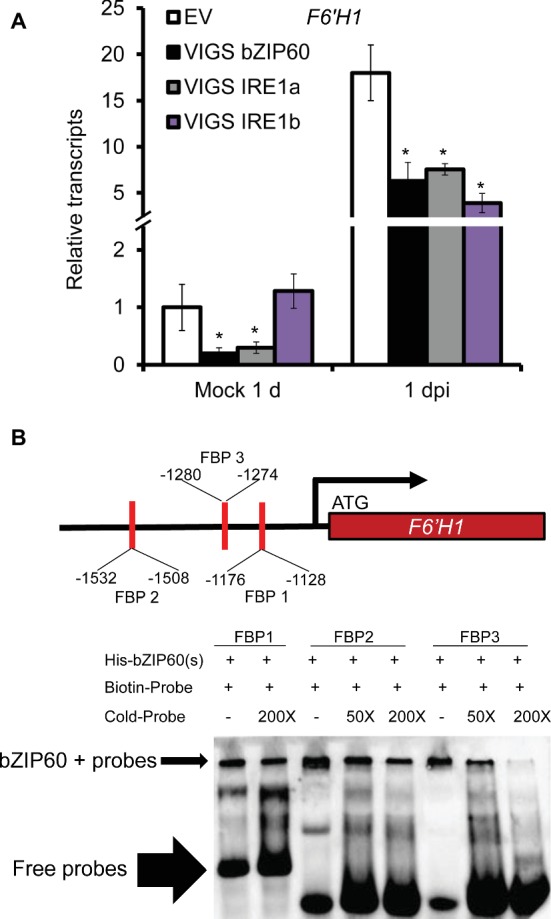
Reduction of *A. alternata*-elicited *F6′H1* expression levels in plants impaired with IRE1-bZIP60 pathway and binding of bZIP60(s) proteins to the probes designed from *F6′H1* promoter. **(A)** Mean (±SE) *F6′H1* transcripts were measured by real-time PCR in five biological replicates of source-sink transition leaves treated with mock infection of *A. alternata* at 1 and 3 dpi. Asterisks indicate the level of significant difference between EV and VIGS plants with the same treatments (Student’s *t*-test: *, *p* < 0.05; **, *p* < 0.01, *n* = 5). All those experiments were repeated three times. **(B)** The spliced form of bZIP60 (His-bZIP60(s)) was purified from Ni-NTA agarose (QIAGEN) beads and incubated with biotin-labeled FBP1, FBP2, and FBP3 designed from *F6′H1* promoter. Unlabeled FBP1, FBP2, and FBP3 were used as the competitor to show binding specificity. The arrows indicate the position of the binding bands and free probes. All those experiments were repeated three times.

As scopoletin and scopolin are important phytoalexins produced in *N. attenuata* against *A. alternata* (Sun et al., 2014; Li and Wu, 2016), we also investigated the gene expression level of the key enzyme gene for their biosynthesis, *feruloyl-CoA 6*′*-hydroxylase 1* (*F6*′*H1*). *F6*′*H1* was highly elicited in EV plants after inoculation, but its expression was dramatically reduced in VIGS *IRE1a*, VIGS *IRE1b*, or VIGS *bZIP60* plants at 1 dpi (Figure 3A), suggesting that scopoletin-based defense was impaired in VIGS plants.

By promoter motif scanning, we found three candidate binding sites of bZIP proteins, namely FBP1, FBP2, and FBP3 in *F6′H1* promoter region, according to TGACTGR, TACGTA, and TGAAAA motifs which were identified previously (Jakoby et al., 2002; Iwata et al., 2008). We investigated whether bZIP60(s) protein could bind to those probes by EMSA. Our results indicated that bZIP60(s) protein could directly bind to all the three biotin-labeled probes (Figure 3B). To see whether the binding was specific or not, excessive unlabeled probes were added respectively and shown to be an effective competitor for each binding (Figure 3B).

### Requirement of JA Signaling in *A. alternata*-Elicited Chaperone Protein Genes and Genes Involved in IRE1-bZIP60 Pathway

**Figure 4:**
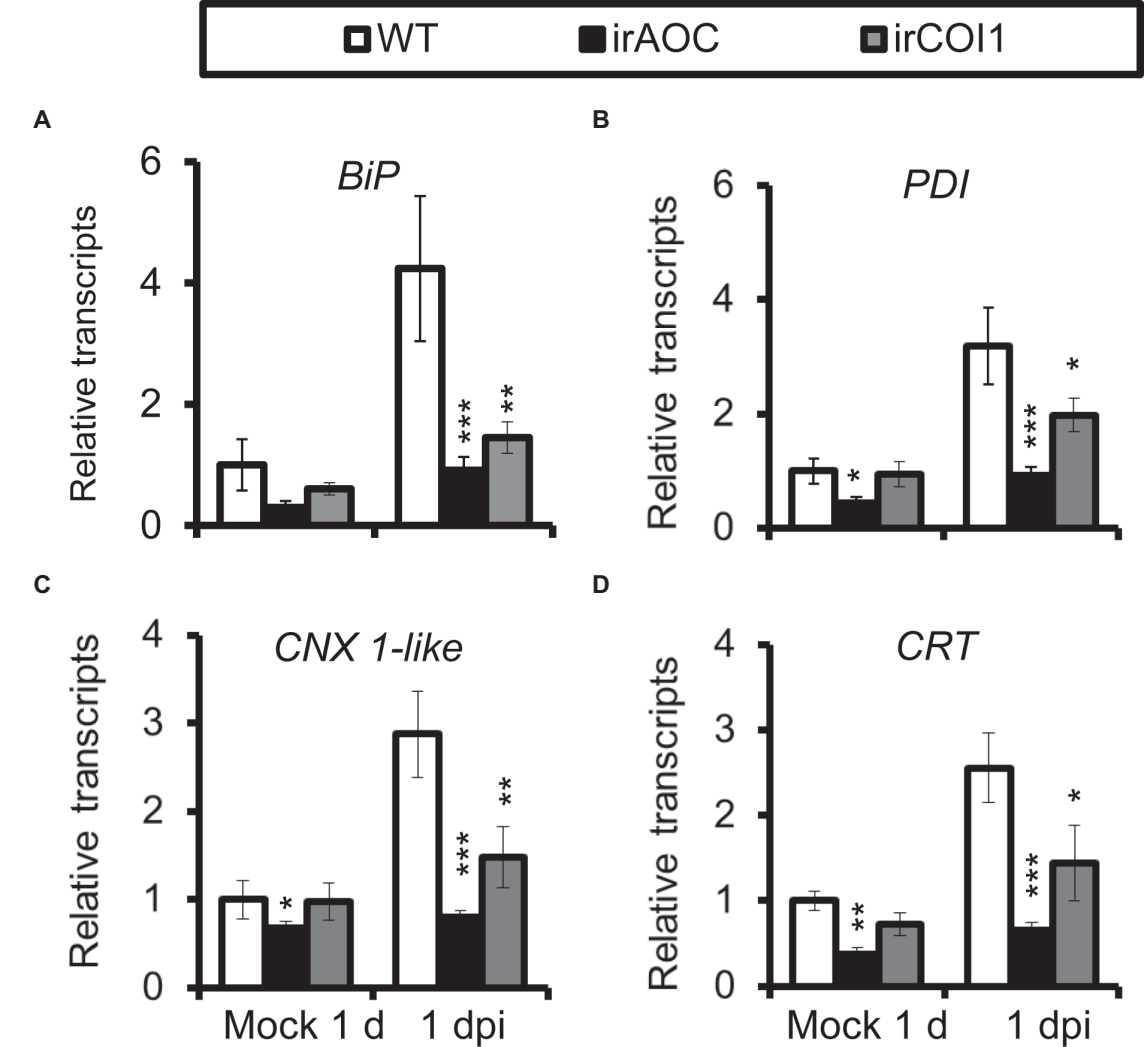
Transcriptional levels of *A. alternata*-induced chaperone protein genes were decreased in JA-deficient or JA-insensitive plants. Mean (±SE) *BiP* **(A)**, *PDI* **(B)**, *CNX 1-like* **(C)**, and *CRT* **(D)** transcripts were measured by real-time PCR in five biological replicates of source-sink transition leaves treated with mock infection of *A. alternata* at 1 dpi. Asterisks indicate the level of significant difference between WT and transgenic plants with the same treatments (Student’s *t*-test: *, *p* < 0.05; **, *p* < 0.01; ***, *p* < 0.005, *n* = 5). All those experiments were repeated three times.

**Figure 5:**
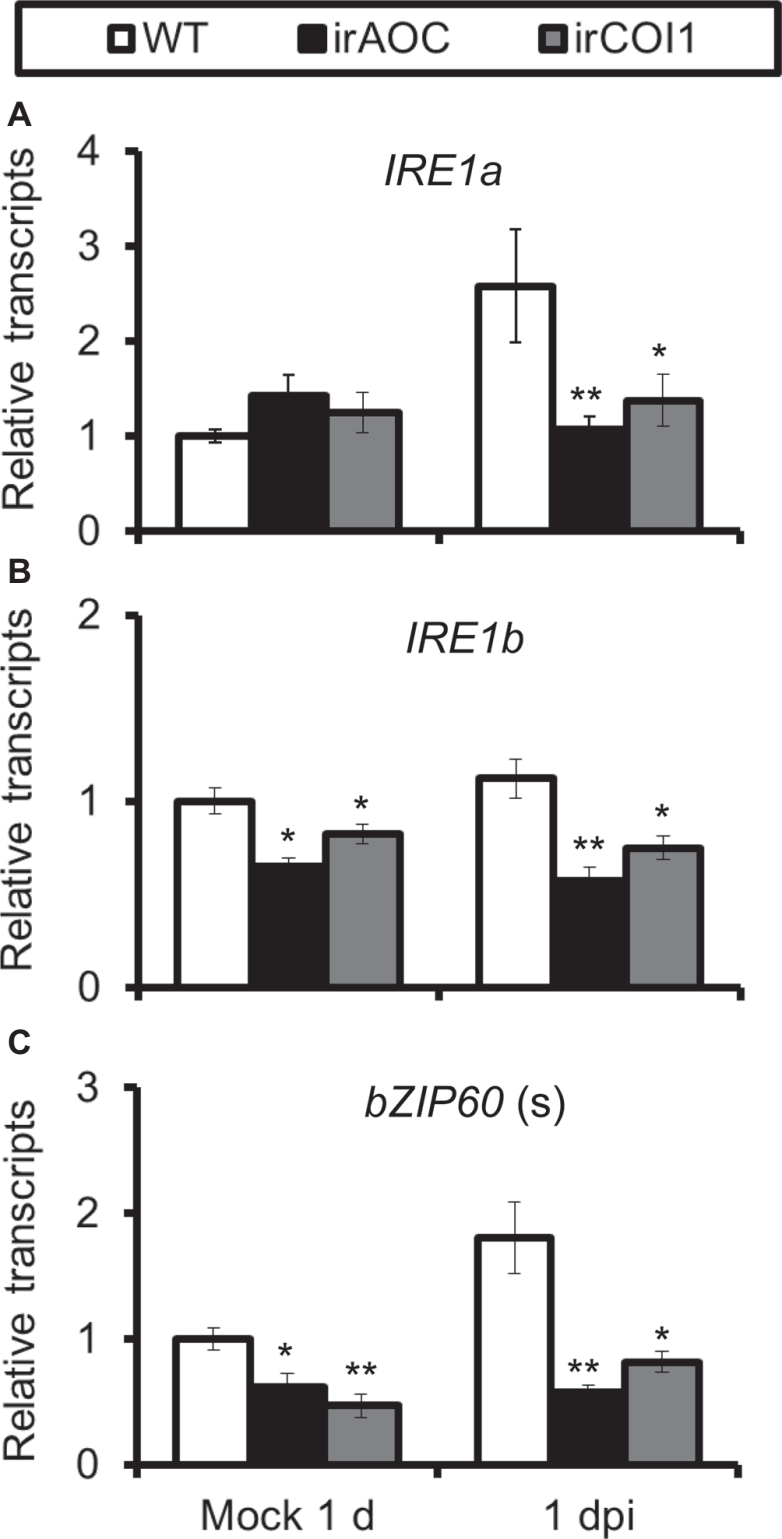
Transcriptional levels of *A. alternata*-induced genes involved in IRE1-bZIP60 pathway were reduced in JA-deficient or JA insensitive plants. Mean (±SE) *IRE1a* **(A)**, *IRE1b* **(B)**, and *bZIP60*(s) **(C)** transcripts were measured by real-time PCR in five biological replicates of source-sink transition leaves treated with mock infection of *A. alternata* at 1 dpi. Asterisks indicate the level of significant difference between WT and transgenic plants with the same treatments (Student’s *t*-test: *, *p* < 0.05; **, *p* < 0.01, *n* = 5). All those experiments were repeated three times.

As JA-deficient (irAOC, plants silenced with *allene oxide cyclase*) and JA-insensitive (irCOI1, plants silenced with JA-Ile receptor *COI1*) plants were highly susceptible to *A. alternata* (Sun et al., 2014), we tried to investigate the role of JA signaling in this *A. alternata*-induced UPR. Compared with WT plants, all four chaperone protein genes decreased their expression levels significantly in irAOC and irCOI1 plants at 1 dpi (Figure 4). Furthermore, the fungus-elicited expression levels of *IRE1a* and *bZIP60*(s) were abolished in irAOC and irCOI1 plants at 1 dpi (Figure 5). Although the gene expression of *IRE1b* was not affected after fungal inoculation, its level was significantly reduced in irAOC and irCOI1 plants at 1 dpi (Figure 5). Those data suggest that JA signaling is required for the elicitation of chaperone protein genes and genes involved in IRE1-bZIP60 pathway.

**Figure 6:**
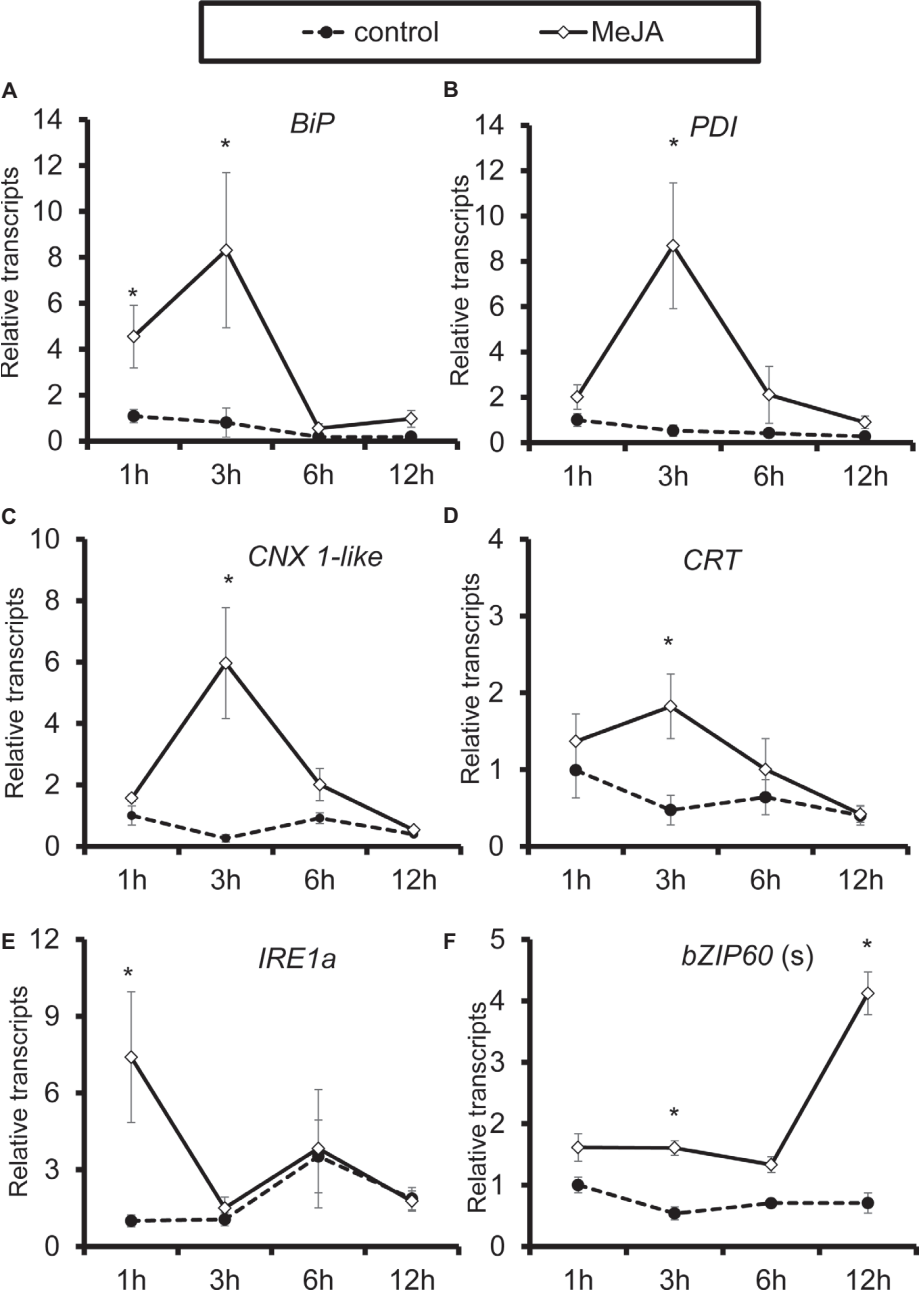
Elicitation of chaperone protein genes and genes involved in IRE-bZIP60 pathway by methyl jasmonate (MeJA). Mean (±SE) *BiP* **(A)**, *PDI* **(B)**, *CNX 1-like* **(C)**, *CRT* **(D)**, *IRE1a* **(E)**, and *bZIP60*(s) **(F)** transcripts were measured by real-time PCR in five biological replicates of source-sink transition leaves treated with mock infection of MeJA at 1, 3, 6, and 12 h. Asterisks indicate the level of significant difference between mock or MeJA treatment (Student’s *t*-test: *, *p* < 0.05, *n* = 5). All those experiments were repeated three times.

When treated with methyl jasmonate (MeJA) exogenously, all genes were significantly induced in *N. attenuata* leaves. All four chaperone protein genes reached their peaks at 3-h treatment, while the highest level of *IRE1a* transcripts was detected after treatment with 1 h, and *bZIP60*(s) at 12 h (Figure 6). These results indicated that MeJA treatment itself is sufficient for elicitation of chaperone protein genes and genes involved in IRE1-bZIP60 pathway.

**Figure 7:**
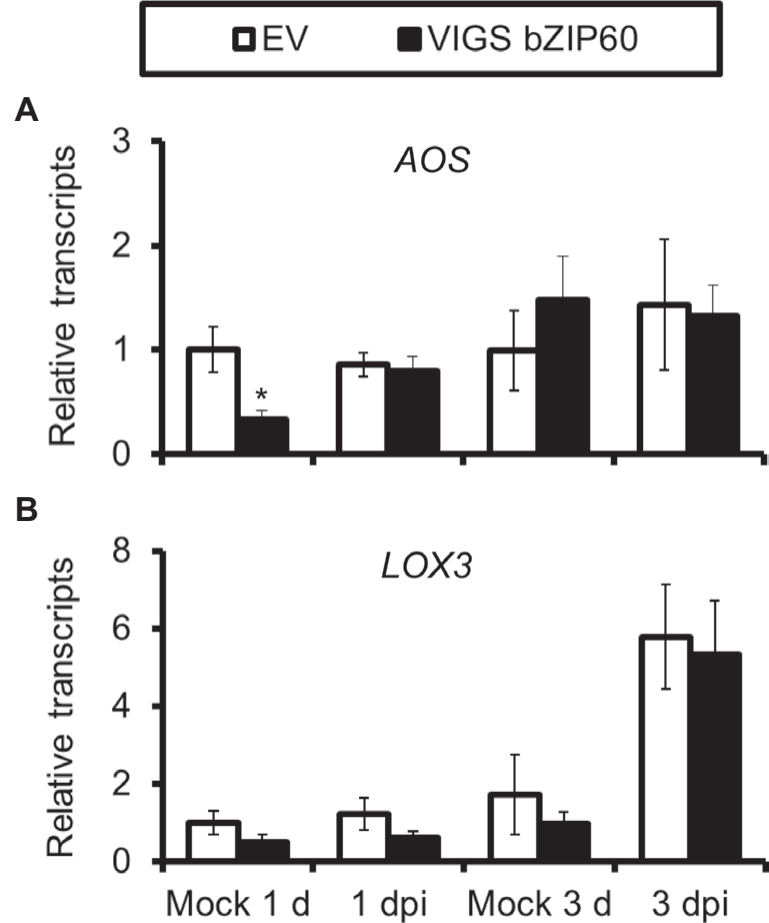
*AOS* and *LOX3* expressions are not affected in VIGS bZIP60 plants. Mean (±SE) *AOS* **(A)** and *LOX3* **(B)** transcripts were measured by real-time PCR in five biological replicates of source-sink transition leaves treated with mock infection of *A. alternata* at 1 dpi and 3 dpi. All those experiments were repeated three times (Student’s *t*-test: *, *p* < 0.05; *n*=5).

We also tested whether or not JA signaling was altered in plants impaired with IRE1-bZIP60 pathway. The transcripts of the key enzyme genes for JA synthesis *LOX3* and *AOS*, which were usually up-regulated after JA elicitation, were not altered in bZIP60-silenced plants (Figure 7), suggesting that JA signaling pathways were not affected in IRE1-bZIP60-silenced plants.

## Discussion

The UPR signaling pathway plays an important role in adaptation to adverse environmental stresses and growth in eukaryotes. It is generally considered that IRE1-bZIP60 branch is conserved throughout metazoans and plants. The *Arabidopsis* genome encodes two IRE1s, AtIRE1a and AtIRE1b that share 41% amino acid identity (Noh et al., 2002). Both AtIRE1a and AtIRE1b are required for the splicing of bZIP60 mRNA (Nagashima et al., 2011), indicating they are functionally redundant. Similarly, two AtIRE1 homologs, IRE1a and IRE1b, were identified in *N. attenuata* genome. They shared 70 and 44% identity to that of *Arabidopsis*, respectively (Supplementary Data 1). The bZIP60 in *N. attenuata* shared 48% amino acid sequence identity to AtbZIP60 (Supplementary Data 1). Although we did not have the direct evidence of this IRE1a and IRE1b catalyzing the unconventional splicing of the mRNA of *bZIP60*, we did observe the existence of spliced form of *bZIP60* by PCR with the specific primers (Supplementary Data 3), which was also confirmed by sequencing. Moreover, the abundance of *bZIP60* (s) expression in either VIGS IRE1a or VIGS IRE1b plants was significantly reduced (Supplementary Data 3). These results indicated that the IRE1a and IRE1b in *N. attenuata* also functioned reluctantly in splicing *bZIP60* mRNA.

Recent reports indicate that IRE1-bZIP60 pathway is involved in plant resistance to pathogens. For example, the expression of *bZIP60* in *N. benthamiana* is up-regulated upon infection with non-host bacterial pathogen (Tateda et al., 2008). IRE1 and its substrate bZIP60 function as a strictly cognate enzyme-substrate pair to control viral pathogenesis in *Arabidopsis* and *N. benthamiana* (Zhang et al., 2015; Gaguancela et al., 2016). In this study, we showed that UPRs were also activated in *N. attenuata* when inoculated with *A. alternata*, as chaperone genes *BiP*, *PDI*, *CNX 1-like*, and *CRT* were all significantly up-regulated after inoculation (Figure 1). Both *IRE1a* and the spliced form of *bZIP60* were also significantly elicited (Figure 1), and clearly the IRE1-bZIP60 pathway was required for plant resistance to this fungal pathogen, as significantly bigger lesions were observed in plants impaired with IRE1-bZIP60 pathway when compared with EV plants (Figure 2).

Why plants impaired with IRE1-bZIP60 pathway were more susceptible to *A. alternata*? It might be due to the delayed up-regulation of chaperone genes which are important to restore proper protein-folding and release ER stress to avoid cell death when the stresses are overwhelming. Among the four up-regulated chaperone genes, *PDI* and *CNX 1-like* showed reduced gene expression levels in VIGS bZIP60 plants at 1 dpi, but they were elicited to levels similar to that of EV plants at 3 dpi (Supplementary Data 2); while *BiP* and *CRT* were induced to the same levels in VIGS bZIP60 and EV plants at both 1 and 3 dpi (Supplementary Data 2). In addition, *F6′H1*, the key enzyme gene of phytoalexins scopoletin and scopolin previously shown to be crucial for *N. attenuata* resistance to *A. alternata* (Sun et al., 2014; Li and Wu, 2016), was found significantly less elicited in plants impaired with IRE1-bZIP60 pathway (Figure 3). Further EMSA experiments indicated that bZIP60(s) could directly bind to all the three biotin-labeled probes designed from *F6*′*H1* promoter. Thus, we concluded that the IRE1-bZIP60 pathway was not only required for releasing the stresses in ER but also regulated chemical defense by direct binding of *bZIP60*(s) proteins to *F6*′*H1* promoter.

**Figure 8:**
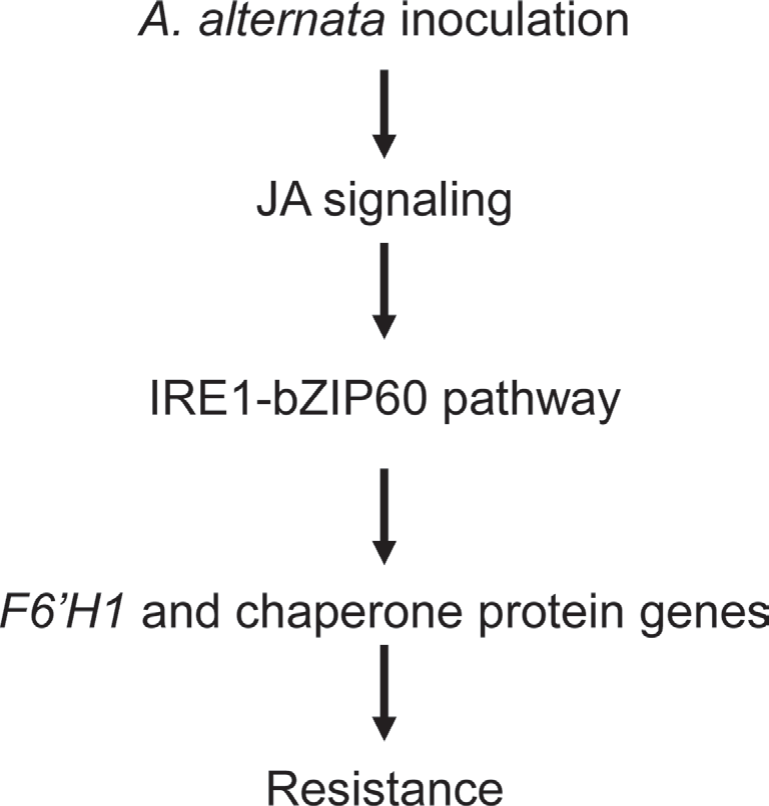
Proposed working model of IRE-bZIP60 pathway in *N. attenuata* resistance to *A. alternata*. After *A. alternata* inoculation, JA signaling pathway is activated, which subsequently regulates *F6′H1* and chaperone protein genes by IRE-bZIP60 pathway.

JA signaling pathway is essential for plant resistance to *A. alternata* (Sun et al., 2014). It regulates a series of defense-related genes; although some of them play a negative role in resistance, like the recently reported patatin-like protein in *N. attenuata* (Cheng et al., 2019), many of them are required for plant resistance to *A. alternata*, for example the pleiotropic drug resistance transporters *NaPDR1* and *NaPDR1-like* (Xu et al., 2018), and *F6*′*H1* (Sun et al., 2014; Li and Wu, 2016). However, whether JA plays a role in UPR is unclear. In this study, we showed that the elicitations of all the four chaperone genes used in this study, *IRE1a*, *IRE1b*, and *bZIP60* by the fungus were dramatically reduced in JA-deficient (irAOC) and JA-insensitive (irCOI1) plants (Figures 4 and 5), suggesting that JA signaling pathway is required for the mounting of UPR. Furthermore, chaperone genes *BiP*, *PDI*, *CNX 1-like*, *CRT*, *IRE1a*, and *bZIP60*(s) were all significantly induced when *N. attenuata* leaves were treated with MeJA (Figure 6). In contrast, the transcripts of the key enzyme genes for JA synthesis, *LOX3* and *AOS*, were not reduced in *bZIP60*-silenced plants (Figure 6), indicating that JA levels are not affected in these plants. Thus our data strongly suggest that JA signaling is located upstream of IRE1-bZIP60 pathway (Figure 8).

Taken together, our study demonstrated that UPR is activated after *A. alternata* inoculation in *N. attenuata*, and the UPR branch, IRE1-bZIP60 pathway, played an important role in plant defense against *A. alternata*. Furthermore, we provided evidence that JA signaling is required for the elicitation of gene expressions of chaperone proteins and genes involved in IRE1-bZIP60 pathway after fungal inoculation.

## Author Contributions

ZX and JW contributed to the conception and design of the study. ZX and JW organized the database. ZX and JW performed the statistical analysis. NS proved the cDNA of irAOC and irCOI1, and cDNA of plants exogenously treated with methyl jasmonate (MeJA), and ZX and JW wrote the manuscript. All authors contributed to manuscript revision, read and approved the submitted version.

### Conflict of Interest Statement

The authors declare that the research was conducted in the absence of any commercial or financial relationships that could be construed as a potential conflict of interest.

## Footnotes

^1^www.sigmaaldrich.com

## Supplementary Material

SUPPLEMENTARY DATA 1 Alignment of the *Arabidopsis thaliana* IRE1a and IRE1b **(a)**, and bZIP60 **(b)** with those of *N. attenuata* protein sequences.

SUPPLEMENTARY DATA 2 *CRT* and *BiP* expressions were not affected but *PDI* and *CNX 1-like* **(b)** were reduced at 1 dpi in VIGS bZIP60 plants. Mean (±SE) *CRT*, *PDI*, *CNX 1-like* and *BiP* transcripts were measured by real-time PCR in five replicates of source-sink transition leaves treated with mock or *A. alternata* at 1 and 3 dpi. (Student’s *t*-test: *, *p* < 0.05; **, *n* = 5) All those experiments were repeated twice.

SUPPLEMENTARY DATA 3 Splicing of *bZIP60* mRNA by IRE1a and IRE1b. **(a)** Mean (±SE) *bZIP60* (s) and *bZIP60* (u) transcripts were measured by real-time PCR in five replicates of source-sink transition leaves treated with mock or *A. alternata* at 1 and 3 day post inoculation (dpi). All transcriptional levels were normalized with a housekeeping gene *Actin II*. Asterisks indicate the level of significant difference between EV and VIGS plants with the same treatments (Student’s *t*-test: *, *p* < 0.05; **, *p* < 0.01, *n* = 5). **(b)** Detection of *bZIP60* spliced form. Unspliced and spliced forms of *bZIP60* were amplified by RT-PCR with primers (XZ141 and XZ142; Supplementary Table 1) from RNA sample extracted from *A. alternata* inoculated leaves at 1 dpi. The PCR products were run on the gel. The band of spliced form of *bZIP60* was pointed by the arrow, and confirmed by sequencing.

SUPPLEMENTARY DATA 4 Photographs were taken of leaves inoculated with *A. alternata* at 5 days post inoculation. Two leaves of EV, VIGS bZIP60, VIGS IRE1a and VIGS IRE1b were provided. Black arrows indicate lesions.

SUPPLEMENTARY TABLE 1 All primers used in this study.
