# Supplementary figures and images for: IRE1-bZIP60 Pathway Is Required for Nicotiana attenuata Resistance to Fungal Pathogen Alternaria alternata
Source: Front Plant Sci. 2019 Mar 19;10:263. doi: 10.3389/fpls.2019.00263 (PMC6434776; doi:10.3389/fpls.2019.00263)

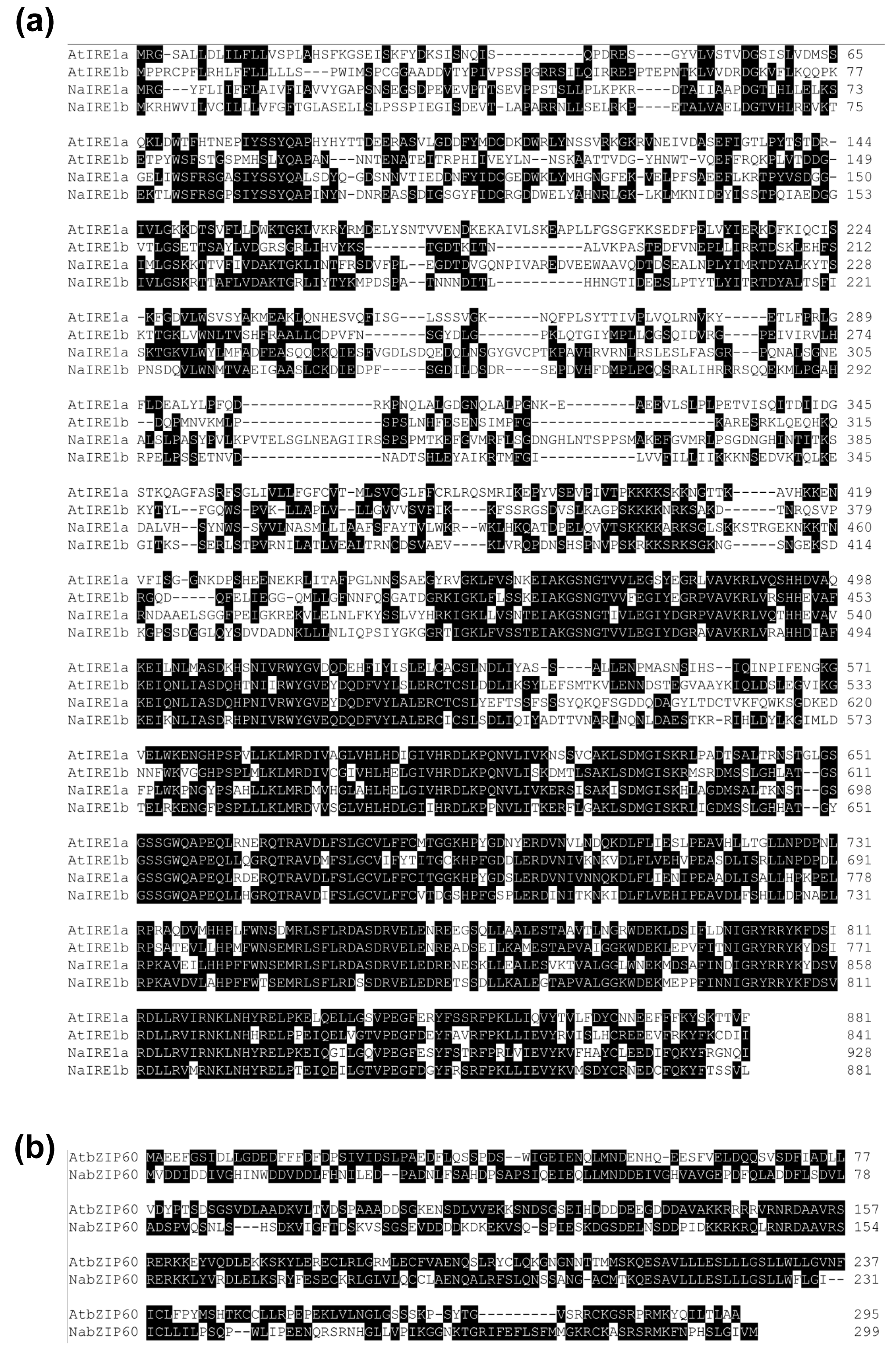

Supplement: SUPPLEMENTARY DATA 1 — Alignment of the Arabidopsis thaliana IRE1a and IRE1b (a), and bZIP60 (b) with those of N. attenuata protein sequences. [file Image_1.TIF]

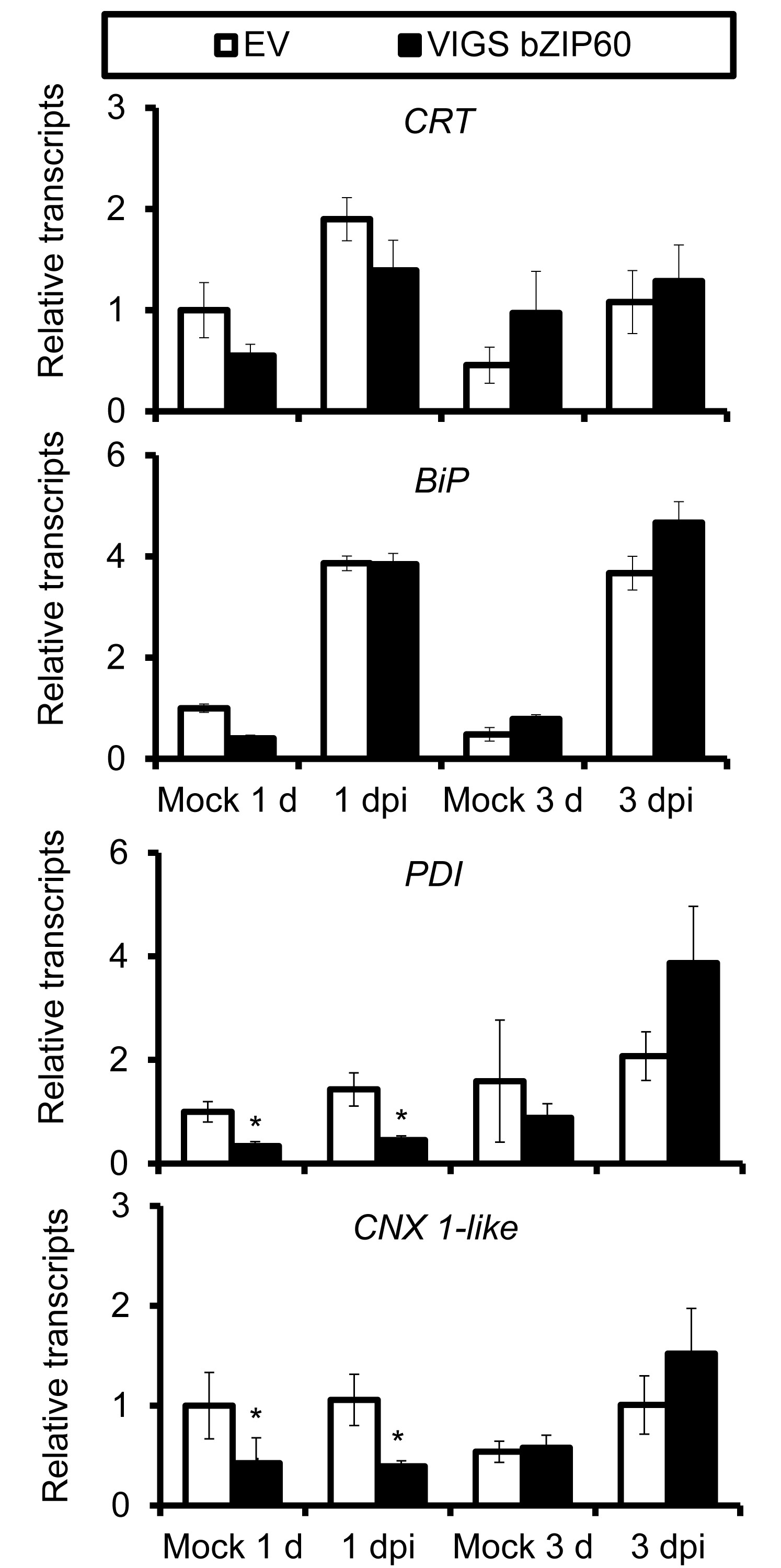

Supplement: SUPPLEMENTARY DATA 2 — CRT and BiP expressions were not affected but PDI and CNX 1-like (b) were reduced at 1 dpi in VIGS bZIP60 plants. Mean (±SE) CRT, PDI, CNX 1-like and BiP transcripts were measured by real-time PCR in five replicates of source-sink transition leaves treated with mock or A. alternata at 1 and 3 dpi. (Student’s t-test: *, p < 0.05; **, n = 5) All those experiments were repeated twice. [file Image_2.TIF]

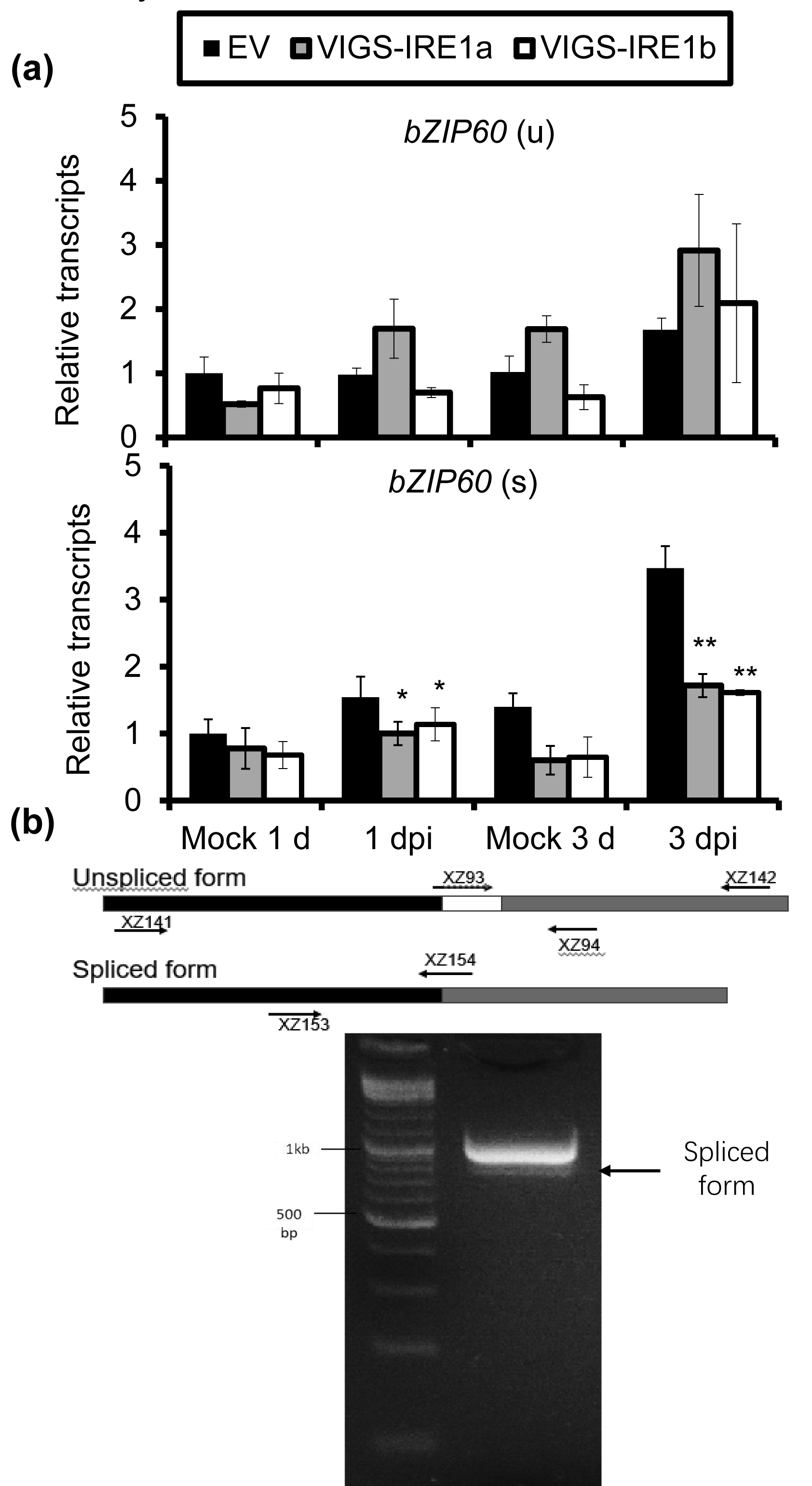

Supplement: SUPPLEMENTARY DATA 3 — Splicing of bZIP60 mRNA by IRE1a and IRE1b. (a) Mean (±SE) bZIP60 (s) and bZIP60 (u) transcripts were measured by real-time PCR in five replicates of source-sink transition leaves treated with mock or A. alternata at 1 and 3 day post inoculation (dpi). All transcriptional levels were normalized with a housekeeping gene Actin II. Asterisks indicate the level of significant difference between EV and VIGS plants with the same treatments (Student’s t-test: *, p < 0.05; **, p < 0.01, n = 5). (b) Detection of bZIP60 spliced form. Unspliced and spliced forms of bZIP60 were amplified by RT-PCR with primers (XZ141 and XZ142; Supplementary Table 1) from RNA sample extracted from A. alternata inoculated leaves at 1 dpi. The PCR products were run on the gel. The band of spliced form of bZIP60 was pointed by the arrow, and confirmed by sequencing. [file Image_3.TIF]

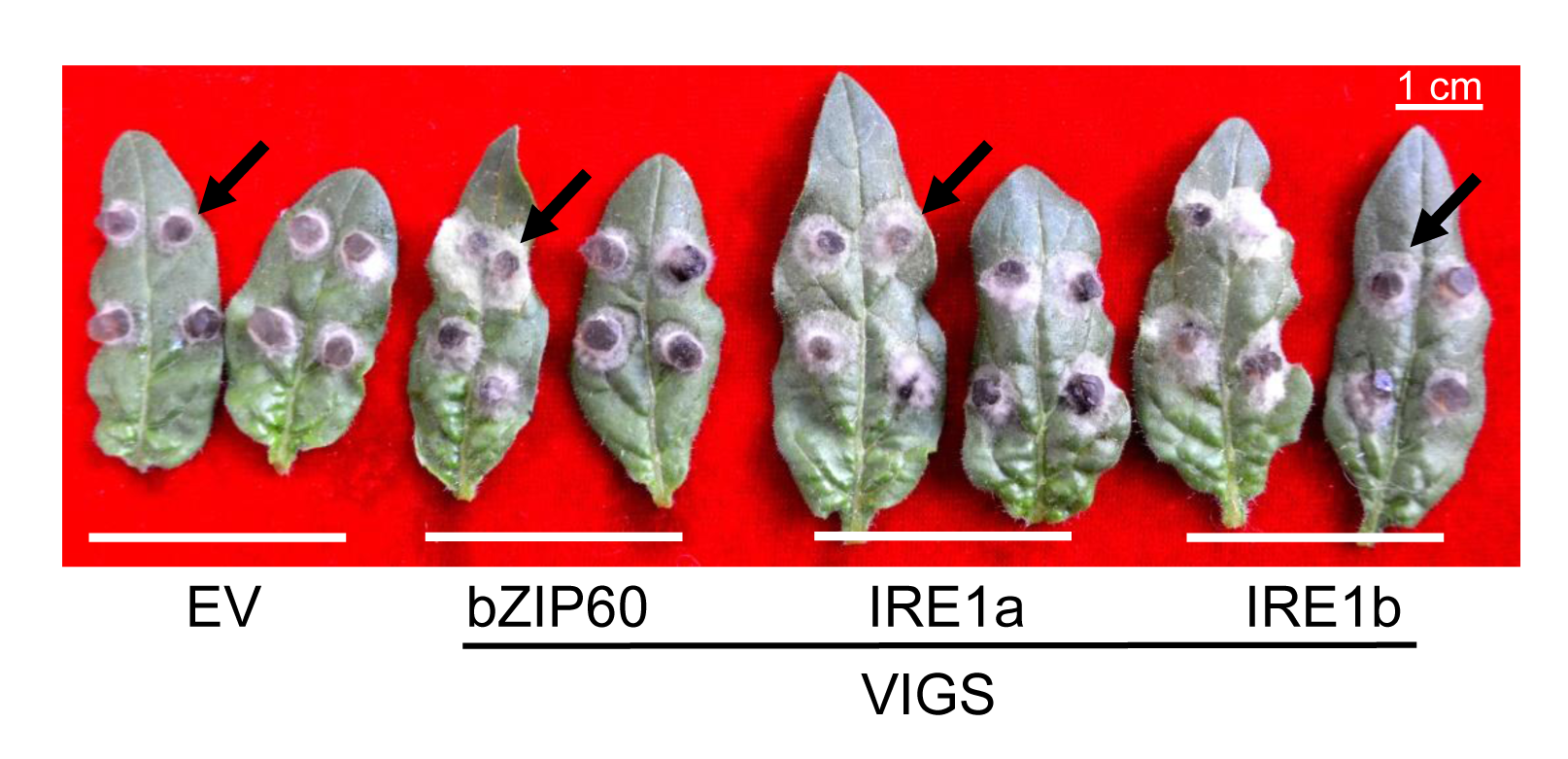

Supplement: SUPPLEMENTARY DATA 4 — Photographs were taken of leaves inoculated with A. alternata at 5 days post inoculation. Two leaves of EV, VIGS bZIP60, VIGS IRE1a and VIGS IRE1b were provided. Black arrows indicate lesions. [file Image_4.TIF]
